# Dimensions of tinnitus-related complaints reported by patients and their significant others: protocol for a systematic review

**DOI:** 10.1136/bmjopen-2015-009171

**Published:** 2016-10-10

**Authors:** Haúla Haider, Kathryn Fackrell, Veronica Kennedy, Deborah A Hall

**Affiliations:** 1 ENT Department, Hospital Cuf Infante Santo—Nova Medical School, Lisbon, Portugal; 2 Nottingham Hearing Biomedical Research Unit, National Institute for Health Research (NIHR), Nottingham, UK; 3 Otology and Hearing group, Division of Clinical Neuroscience, School of Medicine, University of Nottingham, Nottingham, UK; 4 Department of Audiovestibular Medicine, Halliwell Health and Children's Centre, Bolton NHS Foundation Trust, Bolton, UK

**Keywords:** QUALITATIVE RESEARCH, STATISTICS & RESEARCH METHODS

## Abstract

**Introduction:**

Over 70 million people in Europe and >50 million people in the USA are reported to experience tinnitus (the sensation of noise in the absence of any corresponding sound source). Tinnitus is a multidimensional concept. Individual patients may report different profiles of tinnitus-related symptoms which may each require a tailored management approach and an appropriate measure of therapeutic benefit. This systematic review concerns the patient perspective and has the purpose to find what symptoms are reported by people who experience tinnitus and by their significant others.

**Methods and analysis:**

This protocol lays out the methodology to define what dimensions of tinnitus-related symptoms patients and their significant others report as being a problem. Methods are defined according to the Preferred Reporting Items for Systematic reviews and Meta-analyses for Protocols (PRISMA-P) 2015 and data will be collated in a narrative synthesis. Findings will contribute to the eventual establishment of a Core Domain Set for clinical trials of tinnitus.

**Ethics and dissemination:**

No ethical issues are foreseen. Findings will be reported at national and international ENT and audiology conferences and in a peer-reviewed journal.

**Trial registration number:**

CRD42015020629.

**Strengths and limitations of this study**

- The protocol addresses the important question about what people (and their significant others) consider to be the problems related to the tinnitus. These domains can be used to inform choice of outcomes to assess therapeutic benefit.
- The review will collate independent evidence from patient stakeholder groups and actively seeks to reflect an international perspective.
- The review has a clearly established purpose and well-defined methods for data collection and synthesis.
- Limitations include an anticipated bias towards the UK and USA where questionnaires assessing the functional impact of tinnitus have predominantly been developed and an exclusion of reported symptoms where they have not been sorted into domain groupings.

## Introduction

Tinnitus is a symptom—a ringing, buzzing or hissing sound perceived in the ears or head. Most cases of tinnitus are subjective, meaning that tinnitus is perceived only by the patient and there is no clinically identifiable source of the sound, and so assessing tinnitus is reliant on self-report measures. Tinnitus has been associated with a diverse range of complaints, including perceived loudness, sleep problems, existence of additional somatic symptoms, effects on daily life and on general health.^1–3^ Tinnitus may also have negative effects on psychological well-being and personal quality of life, as well as a societal impact in terms of social withdrawal, impaired work performance and suchlike.^4^ ^5^ These examples illustrate how tinnitus is a multidimensional concept. Each of these complaints has the potential to be defined as a domain: a distinct element (or dimension) of tinnitus such as how loud or how emotionally distressing a patient may find his or her tinnitus. In line with other chronic health conditions, these domains can be construed within the WHO's biopsychosocial model of disability relating to impairments, activity limitations and participation restrictions, as well as the environmental factors which affect these experiences.^6^

Self-report measures are used in clinical practice and in research to identify specific areas of a patient's life that are affected by tinnitus (informing the diagnosis), as well as to monitor a patient's progress with a particular treatment (determining the evaluation). However, there is growing acknowledgement that the heterogeneous nature of tinnitus complaints makes clinical research and outcome measurement difficult. For example, a compilation of the multi-item tinnitus questionnaires that have been published over the past few decades indicates at least 29 different instruments. These all purport to measure tinnitus ‘severity’, but do so using different questions, rating scales and subscales. Mostly they have been developed for clinical intake assessment to facilitate ‘doctor–patient’ decision-making about treatment goals and options. Few have been optimised for the evaluation of treatment-related change.^7^ ^8^

Box 1 reports a list, and presents these instruments in chronological order. It is probably fair to say that none of the existing questionnaires covers all of the domains of tinnitus-related complaints. Questionnaire developers draw on clinical experience, but provide limited information in their publications on precisely how they established that the included domains and items are important to patients. There is no current consensus. Some questionnaires are clearly targeted towards the measurement of distinct domains (eg, Fear of Tinnitus Questionnaire;^28^ Self-Efficacy for Tinnitus Management Questionnaire^29^), whereas others are clearly targeted towards the measurement of the overall concept of tinnitus severity (eg, Tinnitus Questionnaire;^9^ Tinnitus Functional Index^31^). Tyler *et al*^34^ have distinguished between primary and secondary effects of tinnitus, claiming that only four primary activities impaired by tinnitus (namely emotions, hearing, sleep and concentration) are relevant for measuring therapeutic changes, at least in such a way that those effects could be specifically attributed to a tinnitus-specific intervention. Patient views and/or opinions were not included in establishing these primary activities and therefore the importance of these domains warrants further investigation. Further research should be expanded to consider reporting of patient-reported complaints, whether they are universal or whether there are cultural differences. Other controversies have been debated. For example, with respect to quality of life, Newman *et al*^6^ proposed that although psychometrically robust measures for tinnitus exist, there is no agreement about what tests should be included in the tinnitus assessment and how studies of health-related quality of life should be conducted.

Box 1 **Multi-item tinnitus questionnaires (first author, date of publication)**

Tinnitus Questionnaire/Tinnitus Effects Questionnaire (Richard Hallam, 1988).^9^

Tinnitus Handicap Questionnaire (Francis Kuk, 1990).^10^

Tinnitus Severity Scale (Robert Sweetow, 1990).^11^

Subjective Tinnitus Severity Scale (Jonathan Halford, 1991).^12^

Tinnitus Reaction Questionnaire (Peter Wilson, 1991).^13^

Tinnitus Severity Grading (Ross Coles, 1992).^14^

Tinnitus Handicap/Support Scale (Soly Erlandsson, 1992).^15^

Tinnitus Severity Index (Mary Meikle, 1995).^16^

Tinnitus Coping Style Questionnaire (Richard Budd, 1996).^17^

Tinnitus Handicap Inventory (Craig Newman, 1996).^18^

Tinnitus Cognitions Questionnaire (Peter Wilson, 1998).^19^

Intake Interview for Tinnitus Retraining Therapy (Margaret Jastreboff, 1999).^20^

Tinnitus Disability Questionnaire (Karoline Greimel, 1999).^21^

Structured Tinnitus-Interview (Wolfgang Hiller, 1999).^22^

Psychological Impact of Tinnitus Interview (Jane Henry, 2001).^23^

International Tinnitus Inventory (Veronica Kennedy, 2005).^24^

Tinnitus Experience Questionnaire (Carol Bauer, 2006).^25^

Chronic Tinnitus Acceptance Questionnaire (Jessica Moreland, 2007).^26^

Tinnitus Acceptance Questionnaire (Vendela Westin, 2008).^27^

Fear of Tinnitus Questionnaire (Rilana Cima, 2011).^28^

Tinnitus Catastrophizing Scale (Rilana Cima, 2011).^28^

Tinnitus Vigilance and Awareness Questionnaire (Rilana Cima, 2011).^28^

Self-Efficacy for Tinnitus Management Questionnaire (Sheri Smith, 2011).^29^

Tinnitus Fear Avoidance Scale (Maria Kleinstäuber, 2012).^30^

Tinnitus Functional Index (Mary Meikle, 2012).^31^

Attention and Performance Self-Assessment Scale (APSA) (Ulli Bankstahl, 2013).^32^

Tinnitus Magnitude Index (Caroline Schmidt, 2014).^33^

Tinnitus Primary Function Questionnaire (Richard Tyler, 2014).^34^

Tinnitus and Hearing Survey (James Henry, 2015).^35^

**Figure 1:**
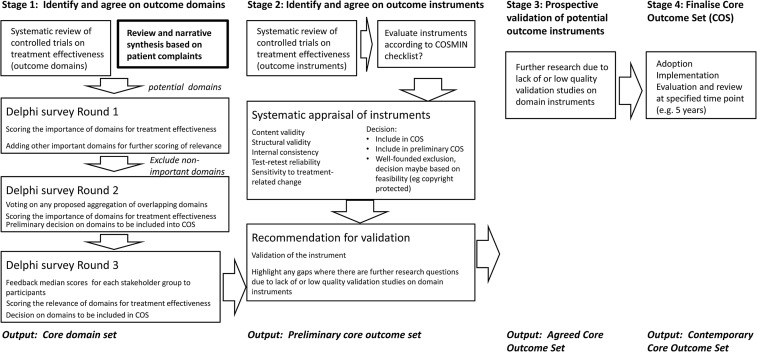
The proposed stepwise roadmap for developing a core outcome set highlighting the component relating to the current systematic review. Adapted from Hall *et al*.^36^

A subgroup (COMiT, Core Outcome Measures in Tinnitus) working under the auspices of the COST Action European network for tinnitus (TINNET, http://tinnet.tinnitusresearch.net/) is currently working collaboratively to establish an international standard for outcome measurements in clinical trials of tinnitus.^36^ Figure 1 illustrates the roadmap for the group. The first deliverable from that roadmap is expected to be a consensus on what outcome domains are essential (ie, core) to be captured in all controlled trials. These core domains will be identified as being important for the design of clinical trials assessing therapeutic benefit because they characterise those tinnitus-related complaints that are relevant to patients and their significant others. The procedures used to identify those core domains will reflect the perspectives of professionals and lay people, alike. This article therefore describes a systematic review protocol of the lay perspective (a first component of stage 1 of that roadmap) that relates to the domains of tinnitus-related symptoms reported by patients and their significant others. Another component of stage 1 includes a companion systematic review of what outcome domains have been evaluated in contemporary clinical trials of interventions for tinnitus.^37^

The opinions of people who experience tinnitus are particularly important because it is this group who tell us what aspects of tinnitus they find problematic and who will experience the benefits and adverse effects of treatments. For example, a recent collaboration with clinical professionals involved people with tinnitus in identifying and prioritising treatment uncertainties and in defining research questions relating to the treatment of tinnitus.^38^ There are many levels in which tinnitus can affect daily lives not only for the person experiencing tinnitus but for those around them as well. Many tinnitus questionnaires ask about the impact of tinnitus on relationships, social activities and work. For example, the Tinnitus Handicap Inventory asks ‘Do you feel that your tinnitus has placed stress on your relationships with members of your family and/or friends?’.^18^ The views and experiences of significant others, close relatives, friends, colleagues and family therefore would provide further insight into the reactions to tinnitus. People should also have opportunities to contribute to any consensus decisions about what are the most important outcome domains to be measured in such research studies. Ideally, one would seek a representative sample for interview to include a diversity of cultural and socioeconomic groups through purposive sampling.^39^ However, acknowledging our limited resources, the COMiT initiative agreed to take a pragmatic approach and to conduct a search to find where others might have already completed such work.

The longer term aim for the findings from this systematic review is therefore to contribute to the development of a core domain set for future controlled trials on tinnitus treatment effectiveness using data collected from people who experience tinnitus and their significant others. Text-based data in particular represents the lay perspective. The objective here is to establish which domains of functioning and disability arising from tinnitus are reported by people who experience the condition because they exert an important impact on their everyday personal life. This objective translates into the primary research question: What are the domains reported by people who experience tinnitus and their significant others which relate to why tinnitus is a problem for them?’.

Secondary research questions include:

- whether patients and significant others have similar or different perspectives;
- whether common clinical phenotypes influence the nature of the reported tinnitus complaints;
- whether health-related comorbidities influences the nature of the reported tinnitus complaints.

## Methods and analysis

Methods are reported according to the Preferred Reporting Items for Systematic reviews and Meta-analyses for Protocols 2015 (PRISMA-P^40^ ^41^). Subheadings correspond to some of the items in the PRISMA-P checklist. The allocation of specific roles to named authors of the review will be made at a later date, and this information will clearly be acknowledged in any subsequent dissemination of findings.

### Eligibility criteria

#### Study designs

Relevant work includes questionnaires, interviews, focus groups and web-based patient discussion forums. Information will be collected from any study type (including surveys, clinical trials and case series). Exclusion criteria are as follows: (1) studies of those medical conditions in which tinnitus is not the primary complaint, such as Ménière's disease, otosclerosis, chronic otitis media and generalised psychiatric comorbidities; (2) any studies whose main focus is on predicting tinnitus severity using regression modelling, rather than measuring tinnitus severity; (3) where individual complaints are reported, rather than having been collected and sorted into domains; (4) review articles and (5) any sources reporting expert opinions, manufacturers' articles, practice guidelines and case reports due to their more limited clinical and scientific value.

#### Participants

We will include men and women who report tinnitus as a primary symptom, as well as including their significant others. The age of the study sample will be ≥18 years. Participants may represent clinical or non-clinical samples.

#### Intervention

The review does not specifically target intervention studies, but may refer to data that are collected as part of the initial assessment. Equally we will consider cross-sectional, non-intervention studies.

#### Comparison

This review does not aim to evaluate specific interventions and as such no comparisons are appropriate or relevant.

#### Outcomes

The review does not specify treatment-related outcomes, but we will consider any types of tinnitus-related problems that are highlighted by patients and their significant others as being potential targets for therapeutic intervention.

#### Timing

All included work will be conducted after January 1980. The rationale is that during the 1980s, many interviews and preliminary questionnaires were administered to relatively large numbers of tinnitus preceding development of a number of the multi-item questionnaires that are now being used to evaluate functional, emotional and other effects of tinnitus.^3^

#### Settings

Articles will be included from research being conducted in any type of setting, such as academic sites where participants are recruited from the general public, non-clinical and occupational groups, and clinical sites including primary and secondary care services.

#### Language

Articles will not be restricted to English language so that we avoid excluding grey literature that is written for a particular lay audience in their native (non-English) language. To facilitate translation, authors will invite members of the EU COST Action TINNET to contribute to the data collection and to summarise in English the relevant information from non-English language documents. At present, the TINNET network includes 29 participating EU countries. For articles written in non-European languages, we will seek to obtain translations by native language speakers recruited from a university setting.

### Information sources

All written documents will be included through the most relevant electronic research databases: PubMed (National Center for Biotechnology Information), Embase (OVID) and CINAHL (EBSCO). Eligible grey literature will include conference papers, undergraduate and postgraduate dissertations, reports from professional organisations and website content. Grey literature databases that will be searched for additional written documents are Open Grey and PsycEXTRA. Dissertations will be searched using DART (for Europe), ProQuest Dissertations and Theses (USA), and Networked Digital Library of Theses and Dissertations (for other databases of named international countries). Conference proceedings will be searched using Cos Conference Papers Index (ProQuest) and Web of Science (Thomson Reuters). Google will be searched using the keywords page by page up to the point at which a page contains no eligible records. In order to seek any further eligible documents for inclusion, we will conduct a manual search of any review articles found through the electronic database search. Tinnitus associations representing patients will also be contacted by email to enquire about commissioned reports and other relevant official documents that may include tinnitus-related complaints. Those tinnitus associations identified by searching Google are listed in box 2.

Box 2 **List of patient associations**

American Tinnitus Association.

Australian Tinnitus Association (New South Wales).

Austrian Tinnitus Association.*

Belgian Tinnitus Association.*

British Tinnitus Association.

Canadian Tinnitus Foundation.

Danish Tinnitus Association.*

Finnish Tinnitus Association.*

French Tinnitus Association.*

German Tinnitus Association.*

Gibraltar Hearing Impaired and Tinnitus Association.

Hungarian Tinnitus Association.*

Irish Tinnitus Association.*

Italian Tinnitus Association.*

Lithuanian Tinnitus Association.*

Netherlands Tinnitus Association.*

New Zealand Tinnitus and Hyperacusis Support Network.

Norwegian Tinnitus Association.*

Polish Tinnitus Association.*

Spanish Tinnitus Association.*

Swedish Tinnitus Association.*

Swiss Tinnitus Association.*

Tinnitus (South Australia).

Tinnitus Association of Victoria.

Turkish Tinnitus Association.*

*Member of the European Federation of Tinnitus Associations.

To ensure literature saturation, we will circulate a bibliography of the included records to tinnitus measurement experts. These are defined as the first author of a published article (known to the authors) that reports the development of a novel multi-item tinnitus questionnaire. First authors are listed in box 1. The manual search and personal contact with tinnitus associations and identified tinnitus experts will be ongoing up to the end of the data collection phase.

### Search strategy

The electronic database search strategy will require ‘tinnitus’ in the title, in conjunction with additional relevant search terms defined as relevant medical subject headings (MeSH) or text words wherever possible. The search terms for PubMed, Embase and CINAHL will be guided by: ‘(tinnitus) AND (problem OR complain* OR symptom)’ OR ‘(tinnitus) AND (patient OR significant other OR partner OR family)’ (table 1). For example, the search strategy for PubMed will be: (((((((((problem[Title/Abstract]) OR complain*[Title/Abstract]) OR symptom[Title/Abstract]) AND (“1980”[Date—Publication]: “3000”[Date—Publication])) AND Humans[Mesh] AND adult[MeSH])) AND tinnitus[Title]) AND Humans[Mesh] AND adult[MeSH])) OR (((((((((patient[Title/Abstract]) OR significant other[Title/Abstract]) OR partner[Title/Abstract]) OR family[Title/Abstract]) AND (“1980”[Date—Publication]: “3000”[Date—Publication])) AND Humans[Mesh] AND adult[MeSH])) AND tinnitus[Title]) AND Humans[Mesh] AND adult[MeSH]). This will be adapted to the syntax and subject headings of the other databases. The authors do not have access to a health information specialist with database searching skills, and so the most experienced researchers will conduct the search.

**Table 1:** Matrix of the search terms for PubMed, Embase and CINAHL

First category (title)	Second category	Third category
Tinnitus	problem (inc. problem identification)	patient (inc. patient care/patient assessment/ patient care planning/patient participation/ patient coding/patient information/patient decision-making/patient preference/ patient satisfaction/patient worry)
	complain*(inc. psychological aspect/consumer)	significant other (inc. family/spouse)
	symptom (inc. symptom assessment/symptom)	partner (inc. interpersonal communication)
		family (inc. family/family attitude/family functioning/family relation/family assessment)
Filter (human/adult) date		

### Study records

#### Data management

HH and KF will be responsible for data management and will have editorial rights. Identified records will be saved into a master file (using Endnote) that will enable records to be tracked through the screening and data collection process. A simple system of record annotation will be implemented to capture reasons for exclusion. Included records will be allocated a study ID code to link each record in the master file with its corresponding full text and data collection sheet.

#### Selection process

Endnote will be used to facilitate screening and remove duplicate records that are being managed within this software system. The first selection step will consider the title information to determine inclusion according to the PICOS and other specified eligibility criteria. All included records will then be reviewed manually to remove any duplicate records, using author names and study title. The second selection step will consider the abstract (or full text for some grey literature sources such as websites) for all potentially relevant records appearing to meet the inclusion criteria or for which there is insufficient information in the title to make a clear decision. Following this, the third selection step will obtain and consider the full text which met inclusion criteria or where there is still any uncertainty in content. The order of initial steps (ie, title screen followed by duplicate removal) is preferred because the search terms are broadly defined and so we predict a large number of ineligible records and also because not all information sources are readily transferred into endnote, for automated duplicate removal. We will adopt the principle that two team members will always perform each key step independently for every record (ie, title screening, full-text screening and data collection). DAH will conduct the key steps for every record, while other individuals may differ. If any discrepancies cannot be resolved, then a third person, an author (HH) not involved with the data screening, will make a judgement on the data entered and act as an arbitrator. Descriptive statistics on the levels of agreement between team members will be reported.

#### Data collection process

Data collection will be guided by an electronic form (excel spreadsheet) that will also be used to collate all the information. To ensure consistency across reviewers, a full set of guidance notes will be produced for the data collection procedure and calibration exercises will be conducted with new members of the review team prior to any individual contribution to this review. The sheet and the guidance notes will be developed and revised through at least two iterations of piloting across several review authors. Data collection will be conducted independently and in duplicate (two people) for every included record. Given the 35-year period of the search, we will not contact the corresponding author by email to seek clarification for any missing data.

### Data items

The data collection sheet will include a list of fields relating to study population, trial design and relevant study findings. Full details are given in box 3. If any information is not reported, then ‘not stated’ will be recorded in the corresponding field.

Box 3 **Data items for systematic review of the domains of tinnitus-related complaints reported by patients and their significant others**

Descriptive checklist:

- Study ID code.
- Record title.
- Name and contact details of corresponding author.
- Country where study is conducted.
- Date of publication (year).
- Aim of study.
- Study population:
  Patients.
  Significant others.
  Other (give details).
- Sample size.
- Age characteristics (mean and SD).
- Tinnitus-related description of study population:
  Duration.
  Intermittent or constant.
  Pulsatile or non-pulsatile.
  Severity.
  Any other subtypes.
- Any other health-related comorbidities in the study population.
- Study type:
  Survey.
  Intervention trial.
  Case series.
  Other (give details).
- Primary method for collecting individual tinnitus-related complaints:
  Questionnaire.
  Interview.
  Focus group.
  Other.
- What specific instructions were given or questions asked?
- Did the authors use open or closed questioning?
  Open.
  Closed.
  Open and closed questions (give details).
- Description of any other relevant data collection methods.
- Primary method for data synthesis.
- Dimensions describing tinnitus-related complaints.
- For each dimension, one example or explanation of what is the underlying theoretical construct.
- Notes (this optional field will be used to record any further comments that may be deemed informative).

### Outcomes and prioritisation

The priority for data synthesis and reporting of findings will be the primary outcome which relates to the domains of tinnitus-related complaints reported by patients and their significant others.

### Risk of bias in individual studies

Given that this systematic review is not concerned with the effects of an intervention for tinnitus, we will not conduct a risk of bias assessment.

### Data synthesis

The main purpose of this systematic review is to identify the domains of tinnitus-related complaints reported by patients and their significant others. A narrative synthesis will be reported using all included records, with information presented in a table to summarise and explain the characteristics and findings. We will seek to preserve the original descriptive labels for domains wherever possible. However, we anticipate that authors of different studies may use different terminology to describe the same underlying theoretical construct. In these cases, we will look carefully at the examples or explanations given by the study authors for each domain of tinnitus-related complaints (see Data items, box 3), and then use this information to cluster together related concepts across studies and report them in grouping table.^42^ For example, Tyler *et al*^34^ refer to ‘concentration’, while Meikle *et al*^31^ refer to ‘cognitive interference’ which includes problems with concentration. For transparency of reporting, all the information on the domain groupings and the associated questionnaire items will clearly be presented in a table so that others can scrutinise our ‘grouping’ decisions. Additionally for narrative text-based data, we will tabulate the evidence for the domain groupings, by providing examples of the narrative text associated with each domain. In this way, each domain that we propose can be traced back to the supporting evidence from all the included studies. A committee including all four coauthors and people with tinnitus (n=4) will examine the choice of words used to describe the labelling of the domains. If there is <70% consensus from the committee that the label is not appropriate for each grouping, then the grouping and labelling will be re-examined in an iterative process.

The narrative synthesis will explore the relationship and findings within and between the included studies as three secondary questions (if there are sufficient data). Main findings will be illustrated by tables.

- To address the secondary research question about whether patients and significant others have similar or different perspectives, we will compile the reported domains of tinnitus-related complaints split according to the study population.
- To address the secondary research question about whether tinnitus ‘subtypes’ influence the nature of the tinnitus complaints that are reported, we will present a table that compiles the reported domains of tinnitus-related complaints split according to common clinical phenotypes of tinnitus (acute/chronic, intermittent/constant, pulsatile/non-pulsatile and severity grading).
- To address the secondary research question about whether a health-related comorbidity influences the nature of the tinnitus complaints that are reported, we will present a table that compiles the reported domains of tinnitus-related complaints split according to the most common comorbidities relating to tinnitus (including mental health problems such as anxiety and depression, hyperacusis and hearing loss).

An exploratory data synthesis will generate a complete list of specific instructions that were given or questions that were asked by the authors to elicit the information from patients and their significant others.

### Confidence in cumulative evidence

Three assessments of the quality of collecting, defining and reporting the domains of tinnitus-related complaints are planned (table 2):

- The first will ascertain whether the participants recruited reflect the heterogeneity of the typical clinical population with tinnitus. A yes/no decision will be based on a weighted composite assessment of the sample size, age characteristics (mean and SD) and any recruitment limitations by subgroup (eg, a specific comorbidity).
- The second evaluate the extent to which the authors used an open questioning format. This is important because closed questioning introduces authors' bias by imposing preconceptions on what sort of tinnitus-related complaints people might consider important.
- We anticipate that a large number of studies will report text-based data, from which themes have been identified. Data analysis of narrative text requires specific skills within the study team and should be reported sufficiently clearly to enable replication. The third quality assessment will therefore assess whether studies report the competencies of those authors conducting the identification of themes. It will also seek to determine the proportion of studies for which the analysis methods have been reported sufficiently clearly to enable replication.

**Table 2:** Quality items for the systematic review of the domains of tinnitus-related complaints reported by patients and their significant others

Quality checklist	Count of records		
Participant sample reflects the heterogeneity of the tinnitus population	Yes (n=)	No (n=)	
Open questioning format	Yes (n=)	No, mixed (n=)	No, closed (n=)
Analysis methods reported sufficiently clearly to enable replication	Yes (n=)	No (n=)	

### Ethics and dissemination

No ethical issues are foreseen. The findings will be reported at national and international ENT and audiology conferences and in a peer-reviewed journal using the Preferred Reporting Items for Systematic Reviews and Meta-Analyses (PRISMA, http://www.prisma-statement.org/). Publication reporting will include the checklist and the flow diagram to depict the flow of information through the different phases of the systematic reviews. All data collected according to the data items will be available on request to the extent that it is not included in the published systematic review article.
